# Fatal Infectious Diseases during Pandemic (H1N1) 2009 Outbreak

**DOI:** 10.3201/eid1711.110429

**Published:** 2011-11

**Authors:** Dianna M. Blau, Amy M. Denison, Julu Bhatnagar, Marlene DeLeon-Carnes, Clifton Drew, Christopher Paddock, Wun-Ju Shieh, Sherif R. Zaki, Infectious Diseases Pathology Branch Working Group

**Affiliations:** Centers for Disease Control and Prevention, Atlanta, GA, USA

**Keywords:** fatal infectious diseases, influenza A virus, H1N1 subtype, staphylococci, streptococci, respiratory infections, influenza, viruses, pandemic (H1N1) 2009

**To the Editor:** Nonpandemic infectious diseases occur with usual incidence during pandemics even though clinical attention is often on the pandemic pathogen. Many of these other infectious diseases share similar clinical signs and symptoms and are sometimes fatal. During the outbreak of pandemic (H1N1) 2009, tissue specimens from case-patients with undiagnosed fatal respiratory illnesses were submitted to the Infectious Diseases Pathology Branch at the Centers for Disease Control and Prevention (Atlanta, Georgia, USA) for evaluation for pandemic (H1N1) 2009 virus infection (*1*).

All respiratory tissue specimens from 450 case-patients received during April 29, 2009–May 5, 2010, were screened by the Centers for Disease Control and Prevention real-time reverse transcription PCR (rRT-PCR) protocol for detection and characterization of swine influenza virus (*2*). Of these, specimens from 250 (56%) tested negative for pandemic (H1N1) 2009 virus and had no other confirmatory or prior influenza testing. Of these case-patients whose specimens tested negative for pandemic (H1N1) 2009 virus, a total of 139 (56%) were male, and the median age was 30 years (range 8 days to 81 years). The median duration from onset of illness to death was 7 days (range 1–40 days). Of the 164 case-patients with available medical records, 127 (77%) had >1 underlying or preexisting medical condition.

When compared with case-patients during the same time who had pandemic (H1N1) 2009 virus infection confirmed by rRT-PCR, case-patients who were not infected with pandemic (H1N1) 2009 virus were more likely to be young (<9 years of age; odds ratio [OR] 2.23, 95% confidence interval [CI] 1.38–3.61) and less likely to be obese (OR 0.6, 95% CI 0.36–0.92) or have asthma (OR 0.33, 95% CI 0.16–0.68). Fever, cough, and shortness of breath were less frequently reported in the case-patients without pandemic (H1N1) 2009.

On the basis of the histopathologic features observed in the respiratory tissues of the case-patients who were not infected with pandemic (H1N1) 2009, along with their clinical and epidemiologic information, the specimens were further evaluated by using special histochemical stains, immunohistochemical tests, and molecular assays. At least l etiologic agent was identified in specimens from 69 (28%) of the 250 specimens (Table). Bacterial pathogens were identified for 44 case-patients; *Staphylococcus aureus* and *Streptococcus pneumoniae* were the most frequently identified. Immunohistochemical tests and PCRs found evidence of viral agents in samples from 26 case-patients. Most of these were seasonal or unsubtypeable influenza A viruses; in a smaller subset, other respiratory viruses were detected, including respiratory syncytial virus and adenovirus. Multiple fungal pathogens were detected in specimens from 2 case-patients.

**Table:** Infectious agents identified in tissue samples from case-patients without pandemic (H1N1) 2009 virus infection, United States, 2009

Agent	No. case-patients*
Bacterial	44
*Staphylococcus aureus*	14
*Streptococcus pneumoniae*	14
*Streptococcus pyogenes*	4
*Streptococcus viridans* group	4
*Leptospira* spp.	2
*Clostridium perfringens*	1
*Capnocytophaga canimorsus*	1
*Haemophilus influenzae*	1
*Legionella* spp.	1
*Neisseria meningitidis*	1
*Pseudomonas* spp.	1
*Rickettsia rickettsii*	1
*Streptococcus agalactiae*	1
Viral	25
Influenza A, unsubtypeable†	10
Influenza A, seasonal (H1/H3)	6
Respiratory syncytial virus	3
Dengue virus	2
Enterovirus	2
Adenovirus	1
Human herpes virus 1	1
Fungal	2
*Aspergillus* spp.	2
*Candida* spp.	2

*For some case-patients, multiple agents were detected. †Subtype not determined by 2 assays, including 1 specific for pandemic (H1N1) 2009 (*2*).

For many of the diseases caused by the pathogens subsequently identified, the clinical features are predominantly respiratory, and many nonspecific manifestations are similar to those of influenza. Nonetheless, >50% of the patients in this study who died of suspected influenza had negative test results for pandemic (H1N1) 2009 virus, and for >25% of these, other infectious causes were detected. Infections other than influenza should be considered during a pandemic and during an endemic influenza season to facilitate the diagnosis of illness and treatment of patients with complications or severe respiratory infections. Although we did not conduct a case–control study, these findings also support the results of other studies that previously reported the demographic characteristics of patients with pandemic influenza infections and the risk factors for severe or fatal pandemic influenza infections (*3*,*4*), especially with respect to obesity (*5*).

Evaluation of tissues collected during autopsy from patients with a suspected infectious process can provide an etiologic diagnosis that was not available from routine premortem and postmortem testing. Other etiologic agents detected in this study included reportable disease agents (e.g., *Rickettsia rickettsii*, *Legionella pneumophila,* dengue virus), vaccine-preventable diseases (e.g., pneumococcal, meningococcal diseases), and zoonotic agents (*Leptospira* and *Capnocytophaga* spp.). These findings underscore the need for autopsies for diagnosing fatal infectious diseases (*6*). They also confirm the need for coordinated surveillance programs that identify deaths potentially attributable to infectious causes, including the unexplained deaths program (*7*) and medical examiner infectious diseases death surveillance program (*8*). Partnerships of medical examiners and pathologists with local, state, and federal public health departments are crucial for detecting and monitoring pandemic diseases and for assessing the scope and magnitude of infectious agents that continuously affect human populations (*9*). These infections often result in sudden or unexplained death; thus, a standardized approach to death investigations is recommended.
